# Surgical management of giant splenomegaly in chronic myeloid leukemia: a challenging splenectomy case

**DOI:** 10.1093/jscr/rjaf485

**Published:** 2025-07-08

**Authors:** Wail Alqatta

**Affiliations:** Department of General and Visceral Surgery, Ibn Sina Hospital, Sanaa, Yemen

**Keywords:** giant splenomegaly, chronic myeloid leukemia, splenectomy, hypersplenism, case report

## Abstract

Giant splenomegaly is an uncommon but significant complication of chronic myeloid leukemia (CML), often causing substantial morbidity. While tyrosine kinase inhibitors have revolutionized CML treatment, splenectomy remains necessary in selected cases, particularly when there is symptomatic hypersplenism, compressive symptoms, or risk of splenic rupture. This case describes a 35-year-old male with chronic-phase CML who presented with progressive abdominal distension, early satiety, and anemia due to massive splenomegaly. Abdominal ultrasound revealed a spleen measuring 40 cm, extending into the pelvis. Despite optimized therapy, symptoms persisted. After multidisciplinary assessment and preoperative vaccination, the patient underwent an open splenectomy. The procedure, though technically challenging, was completed without complications. Histopathology confirmed leukemic infiltration of the spleen. The patient’s postoperative course was uneventful, with no adverse events. This case underscores splenectomy as a viable and effective therapeutic option in selected CML patients with symptomatic giant splenomegaly.

## Introduction

Chronic myeloid leukemia (CML) is a hematologic malignancy characterized by a clonal proliferation of myeloid cells due to the presence of the BCR-ABL1 fusion gene, a consequence of the Philadelphia chromosome translocation t (9;22) (q34; q11) [1]. This genetic alteration leads to constitutive tyrosine kinase activity, driving unregulated cell growth and impaired apoptosis. The introduction of tyrosine kinase inhibitors (TKIs) has significantly altered the clinical course of CML, allowing many patients to achieve long-term remission and near-normal life expectancy [2].

Despite these therapeutic advances, splenomegaly remains a common clinical feature, particularly in patients presenting in the chronic or accelerated phases of the disease. Giant splenomegaly—typically defined as a spleen exceeding 20 cm in length or weighing ˃1000 g—can result in considerable clinical symptoms including abdominal distension, early satiety, and mechanical discomfort, in addition to hematologic complications such as anemia and thrombocytopenia due to hypersplenism [3, 4].

In selected cases, splenectomy is indicated for palliation of symptoms, management of complications such as infarction or rupture, or preparation for stem cell transplantation [5]. However, the procedure in the setting of massive splenic enlargement is associated with significant technical challenges and perioperative risks. These include increased intra-abdominal pressure, distorted vascular anatomy, and heightened risk of bleeding due to friable parenchyma and extensive neovascularization [6].

This report highlights a challenging case of splenectomy in a patient with CML and massive splenomegaly. It underscores the critical importance of thorough preoperative assessment, interdisciplinary collaboration, and meticulous intraoperative planning to optimize surgical outcomes in this rare and complex clinical situation.

## Case information

A 35-year-old male with CML for 8 years, previously treated with chemotherapy, presented with progressive symptoms of massive splenomegaly. He reported persistent left upper quadrant pain, epigastric discomfort, nausea, dyspepsia, constipation, and generalized malaise, significantly impacting daily activities.

Clinical examination revealed severe abdominal distension and a spleen palpable beyond the left costal margin. Ultrasound confirmed splenomegaly with a spleen measuring over 40 cm, mild hepatomegaly (16 cm), and no focal lesions. Laboratory tests showed anemia (Hb 7.1 g/dL), elevated white blood cells (409 × 10^9^/L), and thrombocytosis (816 × 10^9^/L). Renal impairment (creatinine 1.8 mg/dL) precluded contrast-enhanced computed tomography.

**Figure 1:**
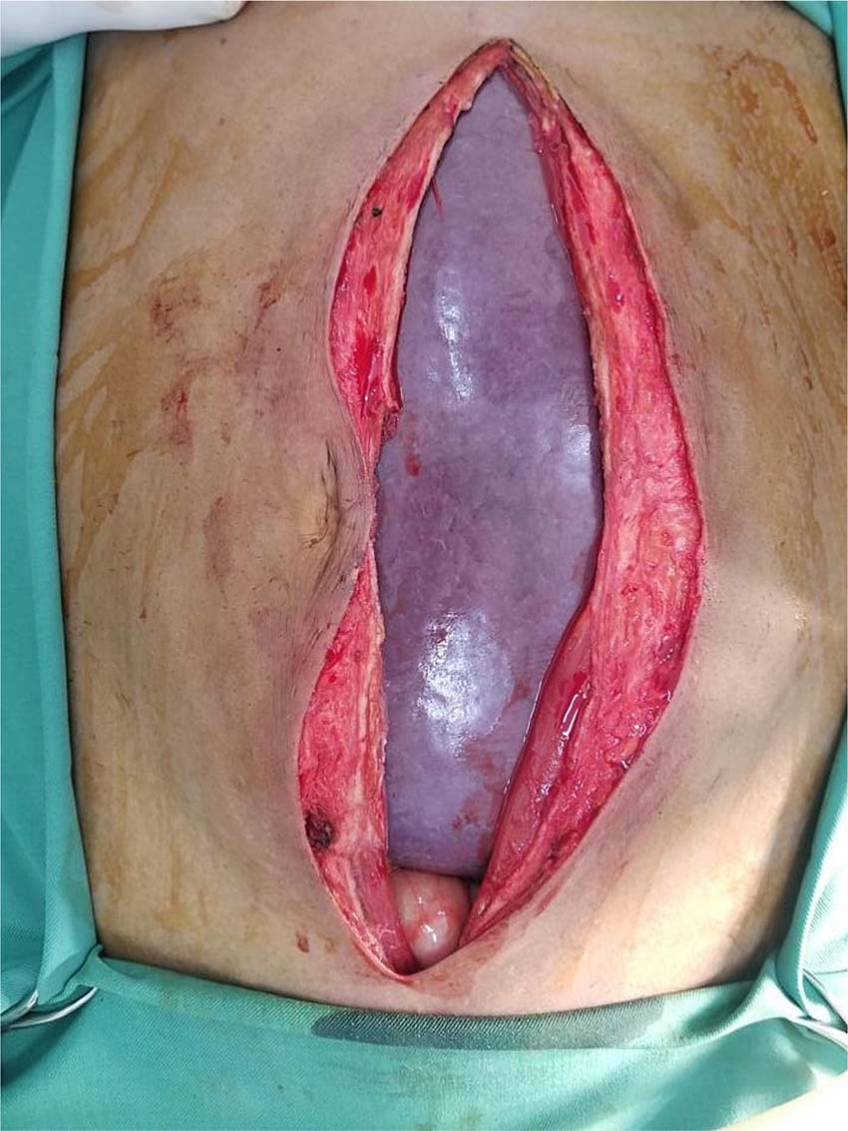
Intraoperative image demonstrating the giant splenomegaly compressing the adjacent intraabdominal organs.

**Figure 2:**
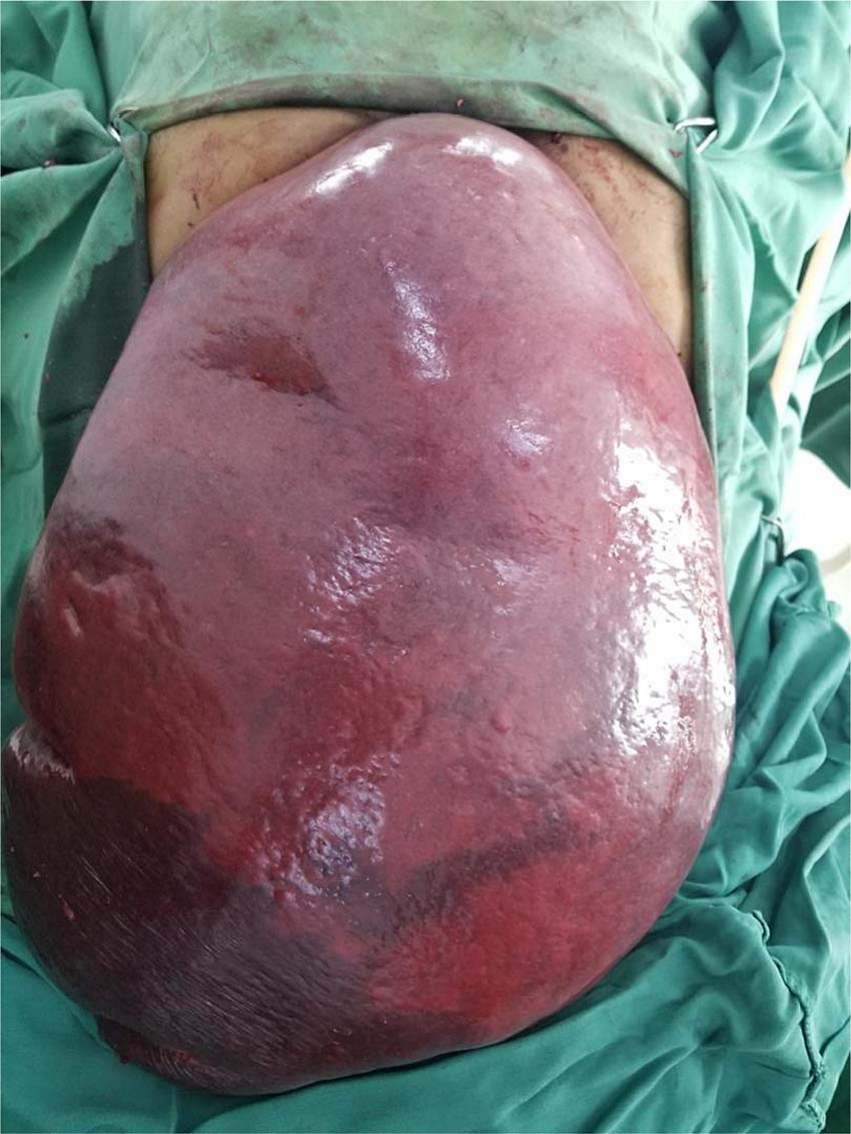
Intraoperative image showing the giant spleen delivered through the peritoneal cavity following careful dissection and adhesiolysis.

**Figure 3:**
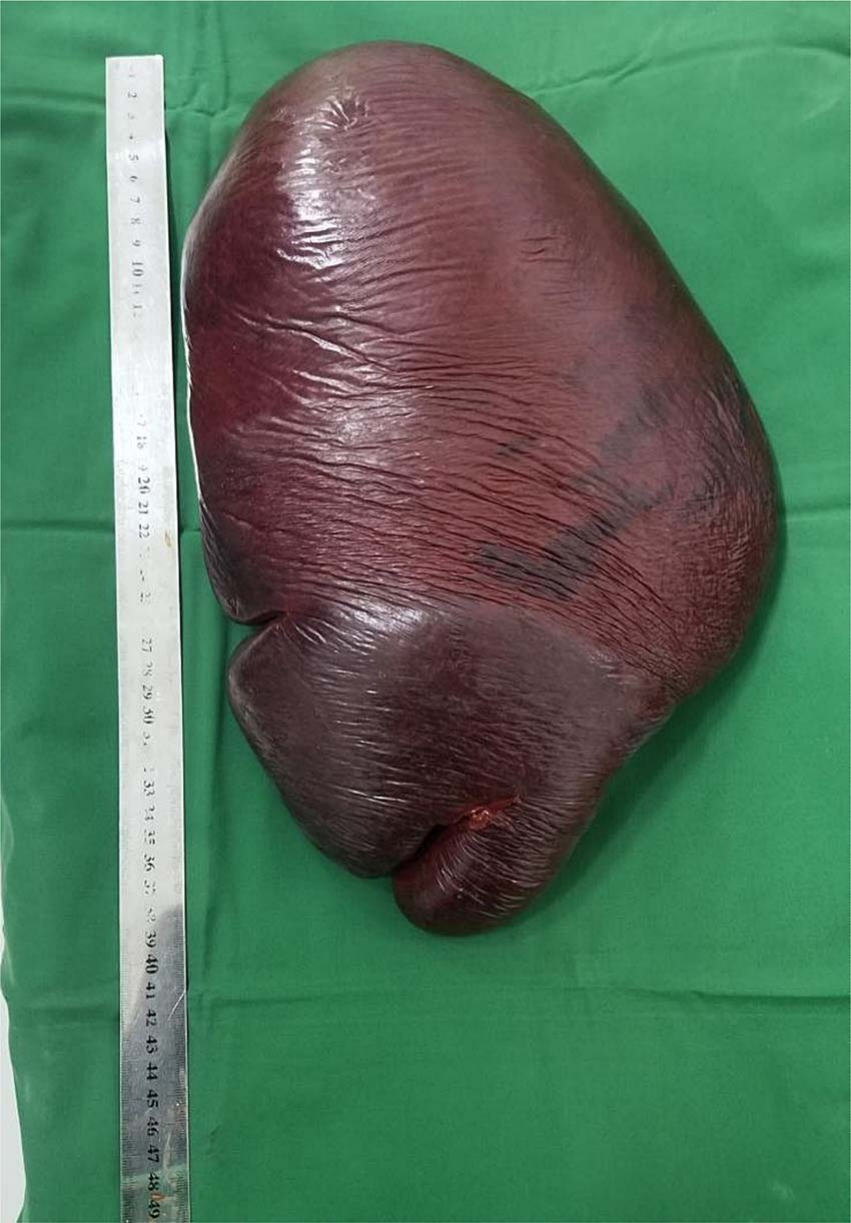
Postoperative image showing the excised giant spleen measuring 40 cm in diameter.

Given hypersplenism and the risk of complications like rupture, the patient was planned for splenectomy. Preoperatively, he received blood transfusions and triple vaccination against *Streptococcus pneumoniae*, *Haemophilus influenzae* type B, and *Neisseria meningitidis*. An explorative laparotomy revealed a giant spleen compressing surrounding organs (Figs 1 and 2). The spleen was successfully removed with minimal blood loss, and histopathology confirmed leukemic infiltration (Fig. 3). The postoperative course was uneventful, and the patient was discharged on the fifth postoperative day.

This case underscores the complexity of performing splenectomy in extreme splenomegaly secondary to CML, requiring careful perioperative planning to minimize risks and ensure a successful surgical outcome.

## Discussion

Massive splenomegaly in CML remains a significant clinical challenge, particularly in cases where TKI therapy is ineffective or disease progression occurs. While TKIs have transformed CML management, some patients still develop symptomatic splenomegaly due to disease progression or resistance to therapy. In such cases, splenectomy may be considered to alleviate symptoms and prevent complications. Splenectomy in CML can provide symptomatic relief, particularly in cases of hypersplenism, abdominal discomfort, or risk of splenic rupture. However, the procedure carries risks, including postoperative complications such as thrombocytosis, venous thrombosis, and infections. A study analyzing outcomes of splenectomy in myeloproliferative disorders reported a 58% complication rate, with thrombocytosis and venous thrombosis being the most common [7]. Therefore, careful patient selection and perioperative management are crucial.

Spontaneous splenic rupture (SSR) is a rare but life-threatening complication in CML, often resulting from leukemic infiltration and splenic infarction. Early recognition and prompt surgical intervention are essential to improve survival [8].

Long-term risks after splenectomy include increased susceptibility to infections, particularly from encapsulated organisms, and a heightened risk of thromboembolic events. A large cohort study involving over 8000 splenectomized patients found significantly elevated risks of pneumonia, meningitis, septicemia, deep vein thrombosis, and pulmonary embolism, underscoring the importance of vigilant postoperative care [9].

Furthermore, patients with large spleens and low in vivo kinase inhibition (IVKI) have been associated with higher incidences of blast crisis and inferior molecular responses, suggesting that spleen size and IVKI are important prognostic factors in CML management [10].

Laparoscopic splenectomy offers benefits like reduced morbidity and quicker recovery. However, in giant splenomegaly (>20–25 cm), it is technically challenging with increased risk of bleeding and conversion. Due to the spleen’s size in this case, open splenectomy was preferred for safety and vascular control [6].

In conclusion, while splenectomy is not routinely indicated in CML, it remains a valuable therapeutic option in selected patients with symptomatic massive splenomegaly or complications such as SSR. A multidisciplinary approach involving hematologists, surgeons, and anesthesiologists is essential to optimize outcomes.

## Author contributions

Dr. Wail Alqatta was responsible for the conception and design of the case report, surgical management of the patient, data collection, literature review, drafting and revising the manuscript, and final approval for publication. Conceptualization, case management, manuscript writing, and final approval of the version to be submitted.
