# Correction to: Surgical management of giant splenomegaly in chronic myeloid leukemia: a challenging splenectomy case

**DOI:** 10.1093/jscr/rjag162

**Published:** 2026-03-06

This is a correction to: Wail Alqatta, Surgical management of giant splenomegaly in chronic myeloid leukemia: a challenging splenectomy case, *Journal of Surgical Case Reports*, Volume 2025, Issue 7, July 2025, rjaf485, https://doi.org/10.1093/jscr/rjaf485

In the originally published version of this manuscript, references 3, 4, 7 and 10 were incorrect and were mistakenly listed as follows:

3. Iannitto E,Tripodo C. Splenic marginal zone lymphoma. *Hematol Oncol* 2005;**23**:55–66.

4. Tefferi A. Splenomegaly in myeloproliferative disorders: clinical features and therapeutic options. *Blood Rev* 2005;**19**:273–86.

7. Tefferi A, Elliott MA, Pardanani A, *et al.* Splenectomy in patients with chronic myeloid leukemia or myeloproliferative diseases: outcomes, complications, and prognostic implications. *Blood* 2007;**110**:2553. https://doi.org/10.1182/blood.V110.11.2553.2553

10. Yeung DT, Osborn MP, White DL, *et al.* Combination of large spleen size and low in vivo kinase inhibition is an early predictor of inferior molecular response and poor outcomes in chronic myeloid leukemia. *Blood Cancer J* 2017;**7**:e540.

This has been corrected, and the references in the published article have been updated as follows:

3. Arber DA, Orazi A, Hasserjian RP, *et al.* International Consensus Classification of Myeloid Neoplasms and Acute Leukemias: integrating morphologic, clinical, and genomic data. *Blood* 2022;**140**:1200–28. https://doi.org/10.1182/blood.2022015850

4. Chapman J, Goyal A, Azevedo AM. Splenomegaly [Internet]. Treasure Island (FL): StatPearls Publishing; 2025 Jan– [updated 2023 Jun 26; cited 2025 Jul 8]. Available from: https://www.ncbi.nlm.nih.gov/books/NBK430907/

7. Tam CS, Pollock RE, Manning J, *et al.* Splenectomy in patients with chronic myeloid leukemia or myeloproliferative diseases: outcomes, complications and prognostic implications. *Blood* 2007;**110**:2553. https://doi.org/10.1182/blood.V110.11.2553.2553

10. Kok C, Saunders V, Dang P, *et al.* Combination of large spleen and low in vivo kinase inhibition is an early predictor of inferior molecular response and poor outcomes in chronic myeloid leukaemia. *HemaSphere*. 2023;**7**:e907256e. https://doi.org/10.1097/01.HS9.0000967516.90725.6e

